# Amorphous/Nanocrystalline High-Entropy CoCrFeNiTi_x_ Thin Films with Low Thermal Coefficient of Resistivity Obtained via Magnetron Deposition

**DOI:** 10.3390/nano13132004

**Published:** 2023-07-04

**Authors:** Maksim Poliakov, Dmitry Kovalev, Sergei Vadchenko, Dmitry Moskovskikh, Philipp Kiryukhantsev-Korneev, Lidiya Volkova, Alexander Dudin, Andrey Orlov, Andrey Goryachev, Alexander Rogachev

**Affiliations:** 1 Merzhanov Institute of Structural Macrokinetics and Materials Science, Russian Academy of Sciences (ISMAN), Chernogolovka 142432, Russia; maxsimpolykovv@gmail.com (M.P.); kovalev@ism.ac.ru (D.K.); vadchenko@ism.ac.ru (S.V.); 2 Institute of Nanotechnology of Microelectronics of the Russian Academy of Sciences, Moscow 119991, Russia; lidiya.volkova.96@mail.ru (L.V.); dudin.a@inme-ras.ru (A.D.); andreyorlov@mail.ru (A.O.); andrei.goryachev@mail.ru (A.G.); 3 Center of Functional Nano-Ceramics, National University of Science and Technology MISIS, Moscow 119049, Russia; mos@misis.ru (D.M.); kiruhancev-korneev@yandex.ru (P.K.-K.); 4 Kotelnikov Institute of Radioengineering and Electronics of the Russian Academy of Sciences, Moscow 125009, Russia

**Keywords:** high-entropy alloy, amorphous structure, magnetron sputtering, thin films, resistors, microelectronic devices

## Abstract

High-entropy alloys are promising materials for novel thin-film resistors since they have high resistivity and a low-temperature coefficient of resistivity (TCR). In this work, a new high-entropy thin-film CoCrFeNiTi_x_ was deposited on a Si/SiO_2_ substrate by means of magnetron sputtering of the multi-component target produced by hot pressing of the powder mixture. The samples possessed a thickness of 130–230 nm and an amorphous atomic structure with nanocrystallite traces. This structure persisted after being annealed up to 400 °C, which was confirmed using X-ray and electron diffraction. The film had a single-phase structure with a smooth surface and a uniform distribution of all elements. The obtained film served for microresistor elaboration, which was produced using the lithography technique and tested in a temperature range from −60 °C up to 200 °C. Resistivity at room temperature was estimated as 2.37 μOhm·m. The results have demonstrated that TCR depends on temperature according to the simple linear law in a range from −60 °C up to 130 °C, changing its value from −78 ppm/°C at low temperatures to −6.6 ppm/°C at 130 °C. Such characteristics show the possibility of using these high-entropy alloy films for resistive elements in contemporary and future micro-electronic devices.

## 1. Introduction

The ever-increasing demand for electronic devices leads to higher requirements for manufactured products. Essential components of microelectronic devices are thin-film resistors produced via magnetron sputtering [1]. In this regard, the requirements for the temperature coefficient of resistance (TCR) are increasing [2]. TCR is one of the key parameters that determine the reliability of an operation. The higher the value of TCR is, the greater the change in parameters occurring during the operation of the device is, and the less accurate it is. The factors that determine TCR include sputtering conditions, annealing temperature, and composition, the last of which is the most important factor [3]. Thus, a search for new compositions of the thin-film resistor elements is in the scope of contemporary materials science.

High-entropy alloys (HEAs) represent a novel class of metallic materials that is often treated as a “new era” in materials science and technology [4,5,6,7,8]. These alloys involve five or more principal components and may possess unique mechanical electrical, magnetic, catalytic, and other properties. Starting from the first publications in 2004 [9,10], several metallurgical techniques, such as casting, mechanical alloying, self-propagating high-temperature synthesis, etc., have been developed for the production of bulk high-entropy alloys and compounds. More recently, high-entropy nanocrystalline or amorphous thin films have demonstrated great potential [11,12].

Among various HEAs, the nanocrystalline composition CoCrFeNiTi_x_ attracts particular attention due to its mechanical [13,14,15,16,17,18] and magnetic [19,20,21] properties, as well as the possibility of using the alloy in 3D additive manufacturing by means of selective laser melting [22] or laser powder bed fusion [23]. Recently, this alloy has been shown to possess good prospects in terms of application in thick corrosion- and erosion-resistant coatings fabricated via laser cladding [24,25].

Currently, the most popular thin-film resistive materials are NiCr [26,27] and TaN [28,29]. Unfortunately, NiCr is easily susceptible to corrosion in a humid environment, and TaN will have a different TCR depending on the position on the substrate despite its higher resistance to oxidation, which makes it difficult to obtain the uniformity of the parameters over the entire wafer. The solution is to carefully control the N content throughout the film, followed by annealing; this is the main reason why TaN cannot completely eliminate NiCr [30].

In the study of [31], NiCrMnZr was synthesized via magnetron sputtering. NiCrMnZr remained amorphous up to 300 °C, then the Ni_7_Zr_2_ phase was formed, and an increase in the Zr content led to a decrease in TCR. The best result of resistance was 510 µOhm·cm under 16.7 at% Zr at 300 °C. In their previous work [32] regarding the Ni-Cr-Si-Al-Ta alloy, Si was found to reduce the TCR, but Ta and Si in the alloy demonstrated poor parameter reproducibility, as well as a low oxidation threshold, resulting in lower operation temperatures.

According to [33], where the influence of the Al content on the electrical parameters of Al_x_CoCrFeNi in the form of a thin film was studied and compared with the bulk material, two regions were identified: the first one, from 27 to 292 °C, and the second, from 292 to 450 °C. In the first region, a low TCR = −5 ppm/°C and a resistivity of 5.36 × 10^−6^ Ω∙m were observed for the Al_0.7_CoCrFeNi alloy with the formation of FCC and BCC structures. The second area demonstrates a strong decrease in resistance due to the formation of the B2 phase.

The decision was to choose Ti instead of Al because Ti has a higher melting point (1668 °C) than aluminum (660 °C) and a higher oxidation threshold, which may be beneficial to the film stability in the case of heat treatment at higher temperatures. Moreover, the HEA films are cheaper than TaN due to the lower price of the constituents. Table 1 summarizes some regimes of thin-film resistive materials synthesis, as well as their resistivity and TCR.

A great potential of using HEA in thin-film resistors was also demonstrated for nanocrystalline CoCrFeNi HEA films produced via magnetron sputtering, which have a high resistivity of 1.35 × 10^−6^ Ohm∙m, hardness of 8.5 GPa, and an elastic modulus of 161.9 GPa [35]. Additionally, CoCrFeMnNi HEA films produced via magnetron sputtering demonstrate an electrical resistivity of 1.3 × 10^−6^ Ohm m and a hardness of 8.5 GPa [36].

It can be concluded that HEA films are promising for research in order to obtain a low TCR, high resistance, and oxidation and corrosion resistance as well, especially considering the possibility of varying the compositions within one alloy.

In our work, we first reported the production of thin nanocrystalline films of CoCrFeNiTi_x_ by means of the magnetron deposition of the multi-metal target on the silicon substrate. The magnetron target was produced from the pure metal powders using hot pressing. Single-phase films with a thickness of ~130–230 nm were produced; the microstructure, atomic/crystal structure, element distribution, electric resistivity (as a function of temperature), and the thermal stability of the films were (everything above is written in the past form) studied experimentally. The results show that the films can be used as resistive elements in various microelectronic devices.

## 2. Materials and Methods

Thin films were deposited through magnetron sputtering on the surface of thermally oxidized monocrystal silicon wafers. The thickness of the SiO layer between the silicon wafer and the film was about 300 nm. The steps from the synthesis of the target to the study of the parameters are described further below.

### 2.1. Target Synthesis

Commercial powders of Co (99.7% pure, ˂70 μm), Cr (98.5%, ˂125 μm), Fe (99.9%, 3 μm), Ni (99.5%, ~150 μm), and Ti (99%, 40 μm) were used as starting materials. The target was prepared according to the composition Co_0.22_Cr_0.23_Fe_0.29_Ni_0.2_Ti_0.06,_ which was characterized as thermally stable HEA [37].

The calculation of $w_{i}$ (wt.%) for each element was performed according to the following formula:

(1) $$w_{i}=\frac{a_{i}\cdot M_{i}}{\sum_{i}^{n}a_{i}\cdot M_{i}},$$

where $a_{i}$—at.% of *i*-th element of the alloy, and $M_{i}$—is the molar mass of the *i*-th element of the alloy. Based on the limitations of the magnetron sputtering setup, the target was a disk, Ø102 × 6 mm with a volume of 49,003 cm^3^. The target density was calculated using the formula:

(2) $$\rho =\frac{1}{\sum_{i}^{n}\frac{w_{i^{\prime \prime }}}{\rho_{i}}}$$

where $\rho_{i}$—density of *i*-th element of the alloy and equals 7819 g/cm^3^. The weight for each element of the alloy was calculated using the formula:

(3) $$M_{i}=w_{i}\cdot \rho \cdot V.\;$$

The total weight of the sample was 383.181 g. The layout of the calculated data is presented in Table 2.

**Figure 1:**
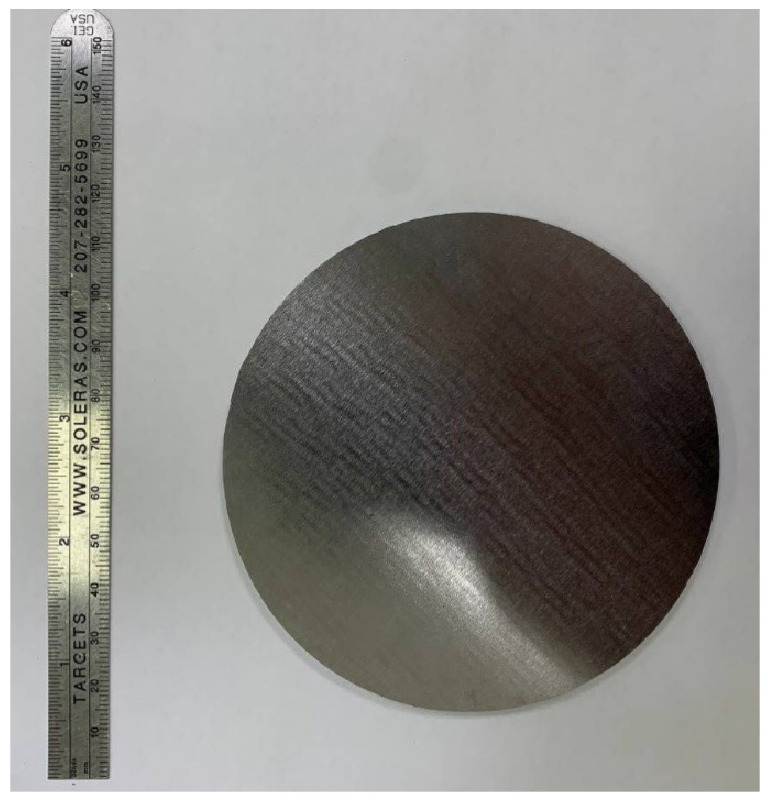
The Co_0.22_Cr_0.23_Fe_0.29_Ni_0.2_Ti_0.06_ target for magnetron sputtering produced via hot pressing.

The powder mixture was prepared in accordance with the calculated sample weight, using a “drum tumbler” mixer and consolidated in a Direct Hot pressing DSP-515 SA press (Dr. Fritsch, Fellbach, Germany) in Ar flow for 60 min at 1000 °C under a pressure of 30 MPa; the heating rate was 30 °C/min. The resulting target is shown in Figure 1.

### 2.2. Magnetron Sputtering of the Film and Preparation of Resistive Elements

Two types of the thin films were deposited using DC magnetron sputtering under the conditions presented in Table 3. Ar 99.9995% purity was used as a working gas. Hydromechanical cleaning was carried out for the substrate. The magnetron power was controlled through the Pinnacle+ power supply (Advanced Energy, Denver, CO, USA).

**Figure 2:**
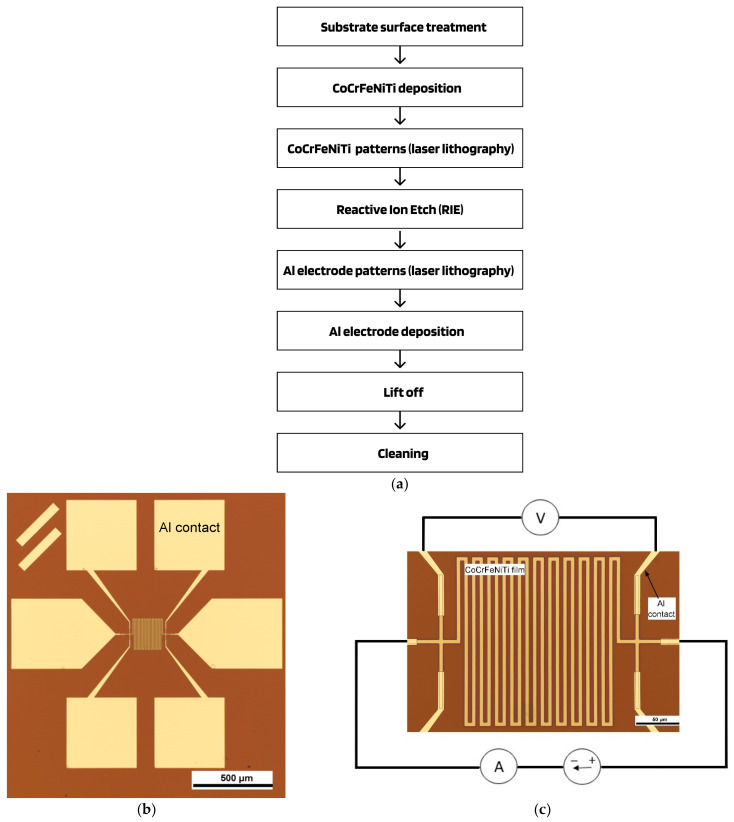
Fabrication procedures of high-entropy CoCrFeNiTi based thin-film resistor structure with Al electrodes (**a**), one of the topologies of test structures thin film resistors (**b**) and the enlarged area of the resistive element with scheme of 4-point resistivity measurement (**c**).

The fabrication procedures of test structures include the deposition of a film via magnetron sputtering, as well as 2 lithography operations to create the topology of structures and pads. The fabrication procedures are shown in Figure 2a. Figure 2b shows an overview image of the structure where the contact regions are made of Al: the central region is a high-entropy CoCrFeNiTi film; an enlarged image of a high-entropy CoCrFeNiTi film as well as a diagram of electrical measurements are shown in Figure 2c.

### 2.3. Analyses of the Films

X-ray diffraction analysis (XRD) of the HEA film was carried out on a PANalytical Empyrean diffractometer (Malvern Panalytica, Malvern, UK) and DRON 3 (NPP Burevestnik, Saint Petersburg, Russia), both with CuKα radiation. A graphite monochromator was installed on the secondary beam to minimize the fluorescent emission of iron-group elements. The survey parameters are presented in Table 4. The analysis of the HEA phase composition was carried out based on the ICDD PDF2 database. To accurately determine the unit cell parameters of the alloy phases, a Si standard (NIST SRM 640D) was added to the powder under study. Full-profile analysis of diffractograms was carried out in the JANA2006 package sequentially using the methods of Le Beyl and Rietveld

XRD measurements at elevated temperature were made using an ARL’XTRA diffractometer (Thermo Fisher Scientific, Waltham, MA, USA)) equipped with a high-temperature chamber HTK2000 (Anton Paar, Graz, Austria). The film sample together with Si substrate was placed in vacuum 10^−3^ Pa on the Cu plate (for a better thermal contact) that was heated up to 400 °C using a tungsten resistive heater. The XRD patterns were obtained at room and high temperature.

A scanning electron microscope (SEM) with focused ion beams and nano-manipulators Helios G4 CX (Thermo Fisher Scientific inc., Waltham, MA, USA) was used for studying the films‘ surface morphology and the preparation of lamella samples for transmission electron microscopy (TEM) analysis. The composition of the sample was studied using energy-dispersive X-ray spectroscopy (EDX); the electron beam current in the scanning transmission electron microscopy (STEM) mode was 1 nA. The High-Resolution Cross-sectional Transmission Electron Microscopy (x-HRTEM) data were obtained from the above lamella with an electron microscope JEM-2100 Plus (JEOL, Tokyo, Japan) operating at 200 kV.

Quantitative element profiling was carried out on a Jamp-9510F (JEOL, Tokyo, Japan) electronic Auger spectrometer (AES). The film under study was etched layer by layer with Ar ions. The parameters of the electron beam: the angle of inclination of the sample relative to the normal to the primary electron beam was 30 degrees. The accelerating voltage of the primary beam electrons was 10 kV, and the beam current was 35 nA. To reduce the primary current density without losing the secondary signal (reducing the effect of artifacts from the electron beam impact on the sample), the electron beam was defocused during the recording of the spectra; the diameter of the analysis region was ~150 μm. The primary current and the degree of the primary beam defocusing were chosen on the basis of the following consideration; the spectrum does not change during the exposure of the sample to the electron beam, which is five-times longer than the spectrum recording time. The parameters of the hemispherical electron analyzer: M4 analyzer operating mode with constant retarding potential and with a relative energy resolution of 0.3%. The parameters of the ion source in profile analysis: the energy of argon ions is 2000 eV; the etching angle is 41 degrees relative to the sample plane. Sputtering during the profile analysis was carried out without sample rotation (the surface of the sample was rather smooth) to reduce the analysis time.

Layer thickness was determined from the average spray rate after measuring the depth of the crater obtained as a result of profile analysis, on a P-7 contact profilometer (KLA Tencor, Milpitas, CA, USA). The recalculation of the intensities of the Auger peaks in concentration was carried out according to the model of a homogeneous distribution of elements in the analyzed layer, taking into account the relative coefficients of the inverse elemental sensitivity [38]. An identification of possible chemical states of an element by Auger spectra was determined by the factor analysis method [39].

Measurements of an electrical resistivity and voltage–current characteristic were carried out in vacuum using semi-automatic probe station PAV200 (Cascade Microtech, Beaverton, OR, USA) in a temperature range from −60 °C to 200 °C, equipped with precise electric measuring source B1500 (Keysight, Santa Rosa, CA, USA) and nano-voltmeter 2182A (Keithley Instruments, Cleveland, OH, USA), see Figure 2c. The resistance was measured at a very small current of less than 10 µA with a variation in polarity and time averaging ~10 s; fluctuations of temperature were less than 0.1 °C.

## 3. Results

### 3.1. XRD Data

**Figure 3:**
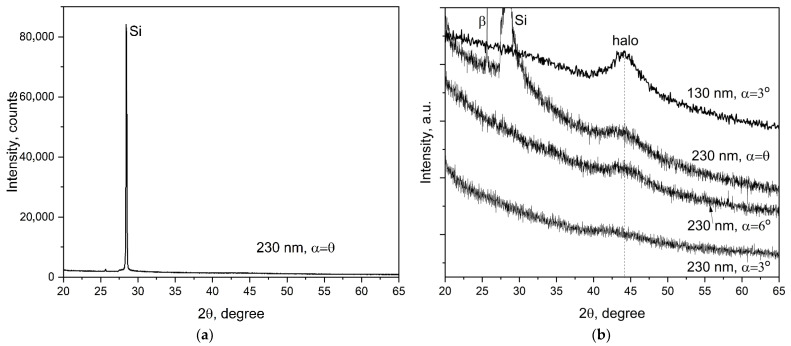
X-ray diffraction patterns of the specimens: (**a**)—symmetric survey (α = θ) of specimen 1; (**b**)—asymmetric survey (α << θ) of specimens 1 and 2.

With the usual symmetrical registration according to Bragg–Brentano, when the incidence angle (α) equals the angle of reflection (θ), the diffraction pattern shows only one sharp peak, 111 Si, from the mono-crystal silicon substrate (Figure 3a). It may be a result of the small thickness of the film or its amorphous structure. A special method with grazing incidence X-ray (small α) was used to reveal reflections from the nanofilms (Figure 3b). Then, the wide halos were observed for both samples in the vicinity of 2θ ~44° (curves 2, 3, and 4 in Figure 3b). A similar halo also became observable in the usual XRD by means of a very close scan of this angle region (curve 1 in Figure 3). This halo can be interpreted as the reflection from very small nano-crystallites that possess an average d-spacing of about 0.207 nm. Thus, we can conclude from the XRD data that the obtained films have a mostly amorphous structure with some presence of nano-crystallites.

### 3.2. Electron Microscopy Data

**Figure 4:**
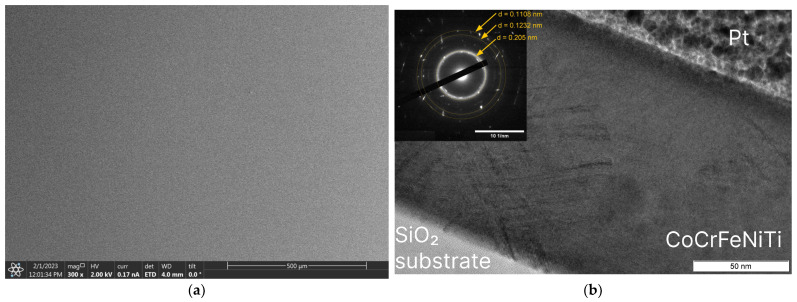
SEM image of the high-entropy CoCrFeNiTi film of specimen 1 (**a**) TEM image of the high-entropy CoCrFeNiTi film with selected area diffraction of specimen 1 (**b**).

The plan view of specimen 1 shows that the film is continuous with a smooth surface (Figure 4a). The bright-field TEM image of the cross-section shows the homogeneity of the film; the film thickness can be measured as 133 nm (Figure 4b), which is in a reasonable agreement with the optical profilometer data (Table 2). The SAED pattern presented in the insert in the upper-left corner of Figure 3b consists of a diffused halo typical for an amorphous phase and several spot reflexes from nano-crystallites. The interplanar spacing of the most intense Debye–Scherrer ring can be calculated from the SAED is d = 0.205 nm. Spot reflexes from separate nano-crystallites can also be attributed to d-spacing 0.1232 nm and 0.1108 nm. The bands on the electron diffraction pattern are associated with stacking defects.

### 3.3. Chemical Composition of the Film

#### 3.3.1. STEM EDX

**Figure 5:**
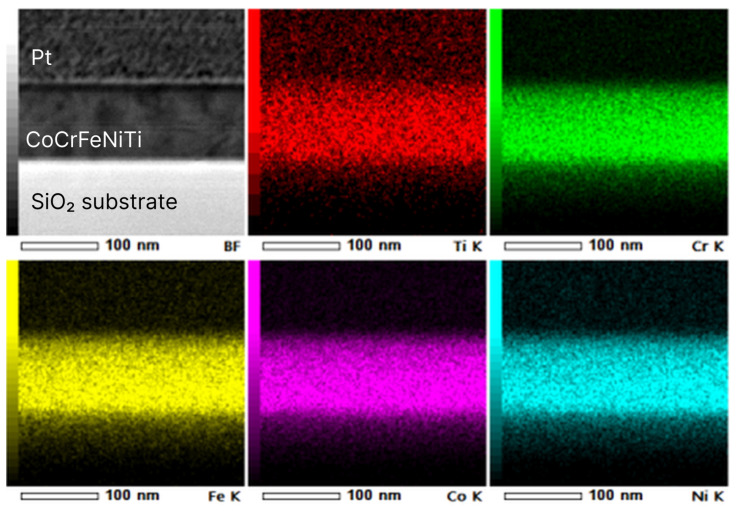
STEM image and X-ray mapping of the cross-section of specimen 1.

The results of STEM and elemental X-ray mapping are presented in Figure 5. The chemical composition of the film, according to EDX analysis (the scan area is 250 × 65 nm), is shown in Table 5. In the quantitative calculation of the chemical composition, possible contamination of the film by oxygen was ignored due to the analytical K-line of O overlapping with the L-lines of Ti and Fe, which made the precise EDX measurement of oxygen concentration hardly available.

It is worth noting that the film differs slightly from the composition of the target (compared with Table 2); it may be the result of different sputtering rates of the elements. The differences are not significant for the film composition; however, they should be taken into account when calculating magnetron target compositions for further research.

#### 3.3.2. Auger Electron Spectroscopy

**Figure 6:**
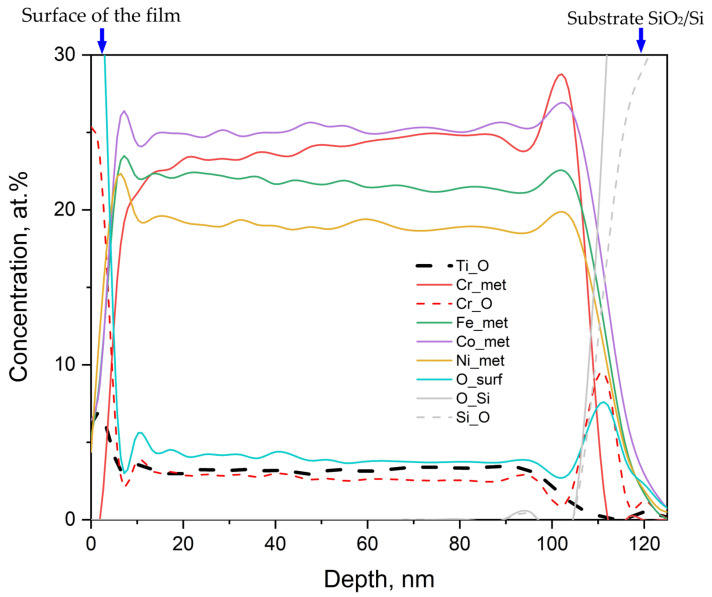
Element concentrations across the film of specimen 1 on SiO_2_/Si substrate. The notation is as follows: the first is the element, the second after the underscore is its chemical bonding or state. For example, Cr_met—metallic chromium, Cr_O—composition of chromium chemically bonded with oxygen. O_surf—oxygen bound to metal.

Figure 6 shows the distribution of element concentrations in sample 1 across the film measured by AES. The left part of Figure 6 represents the film surface; the right part is the substrate. The film surface corresponds to zero etching depth: starting from 110 nm, SiO_2_ begins.

It was possible to reliably identify two forms of the O peak: in metals and in SiO_2_. Unfortunately, the oxygen peaks for different metals are not separated. Oxygen is present in the film: it is bound to Ti and Cr. “Oxidized” iron is present only on the surface; Ni and Co peaks are only from the metal phase. Metallic Ti was not found in the film at all. The surface is clean, and even on the surface, the signal from hydrocarbons is at the noise level. Moreover, it is also absent at the film/substrate interface. Therefore, this peak is not taken into account in the profile. The average values of the quantitative composition obtained by the Auger method in the etching range from 20 to 90 nm are presented in Table 6.

### 3.4. Thermal Stability of the Film

**Figure 7:**
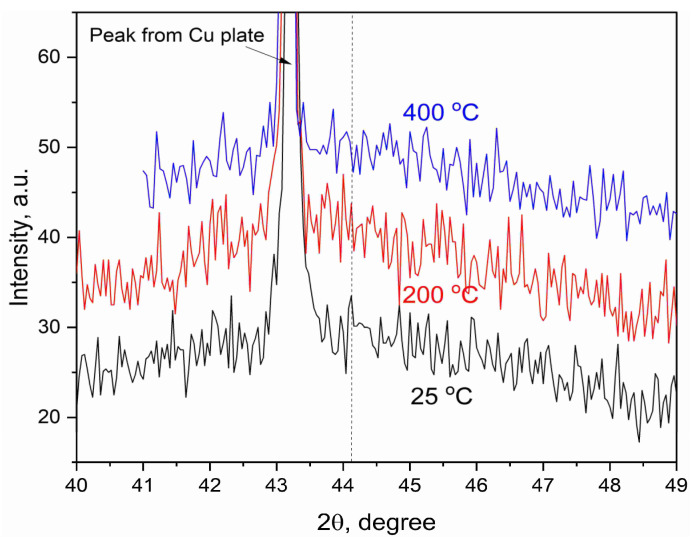
High-temperature XRD of the film of specimen 2. The dotted line indicates the expected position of the most intensive XRD peak (111) for crystalline CoCrFeNiTi alloy [37].

Thermal stability of the film was tested by means of high-temperature X-ray diffraction. The pieces of the Si substrate with deposited HEA film were placed on a Cu hot plate and heated up to 400 °C with continuous registration of the XRD patterns. As shown in Figure 7, the wide halo can be observed at all temperatures along with the peak of Cu. This halo has the same position and width as the initial as-deposited films (see Figure 2b) and they remain stable at elevated temperatures. Thus, we can assume that the amorphous/nanocrystalline structure of the film persists at least up to 400 °C.

### 3.5. Electrical Properties of the Thin-Film Resistors

**Figure 8:**
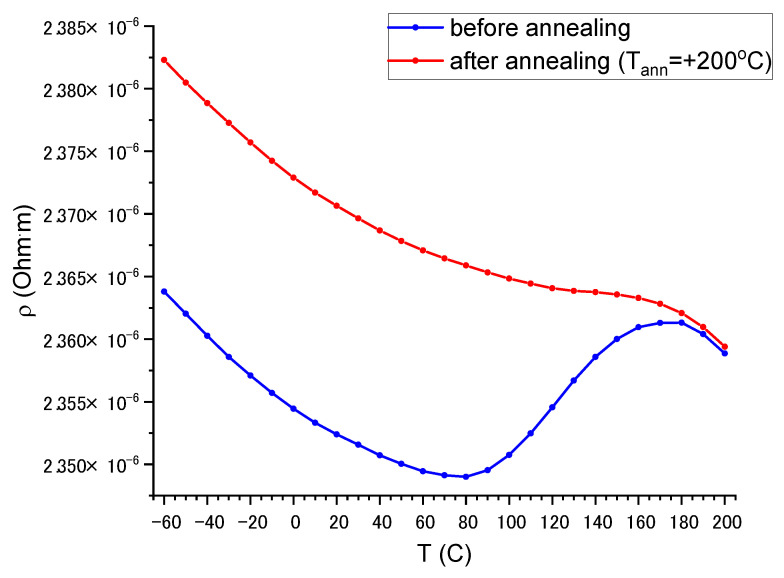
Temperature dependence of electrical resistivity of specimen 1.

Electrical resistivity of the as-produced resistors during the first heating test decreases in a temperature range from −60 °C to 80 °C, then decreases in a range of 80–180 °C and starts to decrease again above 180 °C (blue curve in Figure 8). Probably, such behavior is related to some relaxation processes taking place in the initial amorphous film or the diffusion of some oxygen atoms from the substrate.

**Figure 9:**
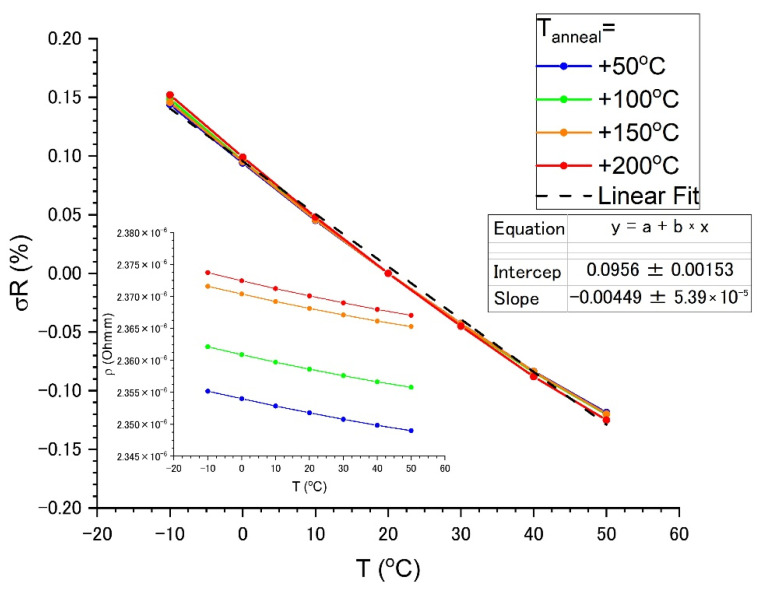
Temperature dependence of the relative change in resistance from the effect of annealing and TCR for specimen 1.

Figure 9 shows the effect of annealing temperature (*T_ann_* = 50, 100, 150, and 200 °C during 3 h) on resistivity in a temperature range from −10 to 50 °C. An increase in the annealing temperature results in a slightly increased (~2%) resistivity. A relative change in the resistivity σR, i.e., the resistivity changes normalized by resistivity at room temperature, does not depend on the annealing temperature. In the considered temperature range, all samples have an almost linear temperature dependence of resistivity with TCR = −45 ± 1 ppm/°C in the practically important region from −10 to 50 °C. Resistivity at room temperature is 2.37 × 10^−6^ Ohm·m.

**Figure 10:**
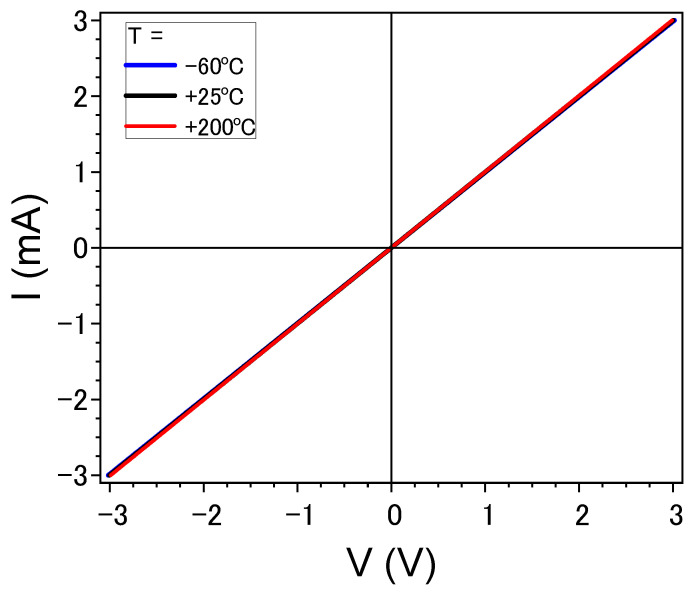
Volt−current characteristics of specimen 1 at different temperatures.

Current–voltage characteristics measured at different temperatures are perfectly linear and do not depend on the temperature, as shown in Figure 10.

In addition to the obtained TCR of the resistive film, the electrical transport properties of the contact resistance with metal (Al) were studied at different annealing temperatures. Thermal annealing not only stabilizes the resistivity of the resistor film but also improves the electrical properties of the contacts, making it possible to obtain a very low-resistance and stable electrical contact between Al electrodes and HEA film (~0.4 Ohm/μm^2^).

## 4. Discussion

The electrical resistivity of the obtained film at room temperature, 2.37 μOhm·m, is much higher than the resistivity of all individual components: Co (0.063 μOhm·m), Cr (0.132 μOhm·m), Fe (0.065 μOhm·m), Ni (0.068 μOhm·m), and Ti (0.54 μOhm·m). This effect is related to the amorphous atomic structure of the film, which results in strong electron scattering. The amorphous structure is confirmed by XRD, TEM, and SAED analyses. In the vicinity of 2θ angle about 44° (Cu-Kα radiation), where the strongest 111 reflection of the FCC HEA solid solution is expected for crystalline bulk material [37], the XRD pattern shows a wide halo (Figure 3b). The maximum intensity of this halo corresponds to interplanar spacing d = 0.204 nm. Atomic interplanar spacing calculated from the SAED (Figure 4b) is d = 0.205 nm. Assuming that this broad maximum corresponds to 111 reflections of disordered and strongly amorphized FCC structure, the lattice constant can be estimated as a = 0.3584 nm.

**Figure 11:**
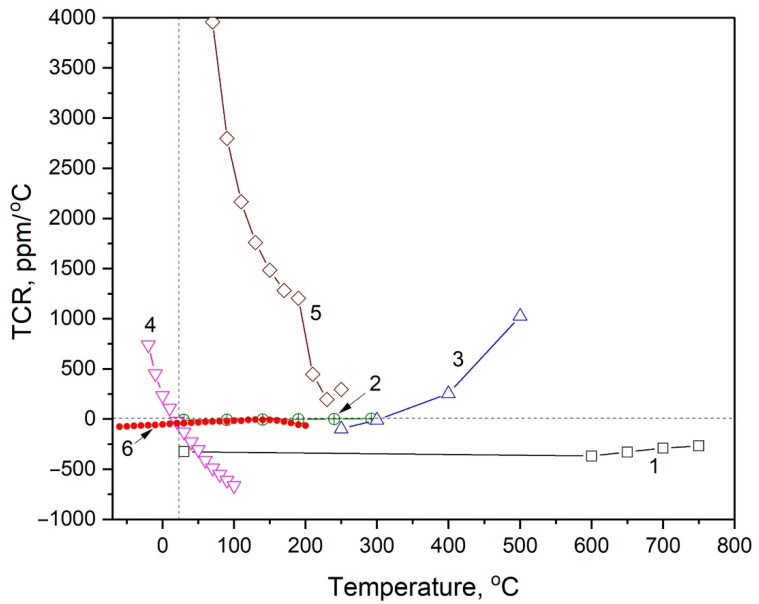
Temperature dependence of TCR for different thin films: 1−TaHf [30], 2−CoCrFeNiAl_x_ [33], 3−NiCrSiAlTa [32], 4−TaN [28], 5−NiCr [26], 6−CoCrFeNiTi_x_ (this work, calculated from the red curve in Figure 8).

The obtained value of resistivity correlates with the resistivity of CoCrFeNiAl_x_ films that can be varied in the range (1.918–5.359) × 10^−6^ Ohm·m by varying the Al concentration [33] and is close to the resistivity of NiCr_x_ films ~2.4 × 10^−6^ Ohm·m [26]. A peculiarity of the CoCrFeNiTi_x_ films is that they possess negative, relatively small TCR in a wide temperature range. A comparison of TCR–temperature dependencies for different films is presented in Figure 11.

In the range of temperature from −60 °C up to 130 °C, variation in TCR with temperature for the obtained film can be described by simple linear dependence

(4) TCR=a+bT, 

where a = −53.863 ± 0.512 ppm/°C and b = 0.3704 ± 0.0076 ppm/(°C)^2^. Modulus of TCR reaches minimum value TCR = −6.6 ppm/°C at the temperature T = 130 °C. Thus, the obtained films possess characteristics comparable with those for the best-known film resistors.

## 5. Conclusions

The new high-entropy film CoCrFeNiTi_x_ was deposited on the Si/SiO_2_ substrate by means of magnetron sputtering of the multi-component target produced by hot pressing the powder mixture. The film has a thickness of 130–230 nm, and the amorphous atomic structure is confirmed by the results of X-ray and electron diffraction. The amorphous structure persists after being annealed at 400 °C. The samples are single-phased with a smooth surface and a uniform distribution of all elements across the films.

Micro-resistors were produced from the deposited films using the lithography technique and tested in a temperature range from −60 °C up to 200 °C. The resistivity at room temperature is 2.37 × 10^−6^ Ohm·m. The temperature coefficient of the resistivity (TCR) depends on temperature according to the simple linear law in a range from −60 °C up to 130 °C, changing its value from −78 ppm/°C at low temperatures to −6.6 ppm/°C at 130 °C. Such characteristics show the possibility of using this high-entropy alloy for resistive elements in contemporary and future microelectronic devices.

## Figures and Tables

**Table 1:** Parameters of resistive films depending on the regimes of synthesis via magnetron sputtering.

Alloy	Power, W (DC)	Working Pressure, Pa	Microstructure	Annealing, °C	Material of Substrate	Ω, 10^−6^ Ohm·m	TCR, ppm/°C	Thikness, nm	Ref.
NiCr	40/70 *	0.67	amorphous	No	SiO_2_ (600 nm)/Si	2.4	±10	100	[26]
NiCr	405	0.85	amorphous with Ni (111)	350	Si	0.74	~6000 (RT)	86	[27]
TaN	700	0.13	amorphous	300	SiO_2_ (50 nm)/Si (100)	13,860	−67 (RT)	198	[28]
TaN	250	0.93	TaN	250	GaAs	4.8	−200 (RT)	600–750	[29]
TaHf	100 **	0.5	Hf_6_O_17_Ta_2_ (200)	No	Si	5.7	−330 (RT)	120	[30]
NiCrSiAlTa	100	0.57	amorphous	300	Al_2_O_3_	22.15	−10 (300 °C)	80	[32]
Al_0.7_CoCrFeNi	100 ***	1.4	FCC and BCC	No	Si (100)	5.36	−5 (RT to 292 °C)	570	[33]
NiCrMnZr	50/30 ****	0.4	amorphous	300	Al_2_O_3_	5.1	53 (from 25 to 125 °C)	80	[31]
NbMoTaW	50	0.67	BCC	No	Si (100)	1.68	None	350	[34]

* 40 W for Ni and 70 W for Cr. ** Two targets were used for magnetron sputtering at 100 W. *** RF magnetron. **** Co-sputtering NiCrMn-50 W (DC) and Zr—30 W (RF). RT—room temperature.

**Table 2:** Calculated weights of elements for target synthesis.

Elements	ρ, g/cm^3^	at.%	AEM	g/mol	wt.%
Co	8.9	22	58.93	12.9646	23.3
Cr	7.19	23	51.996	11.95908	21.5
Fe	7.874	29	55.845	16.19505	29.1
Ni	8.904	20	58.6934	11.73868	21.1
Ti	4.54	6	47.867	2.87202	5.2

**Table 3:** Magnetron sputtering parameters and film thickness.

Name	Power, W	Working Pressure, Pa	Duration, s	Working Gas	Thikness, nm
Specimen 1	500	0.1	75	Ar	130
Specimen 2	500	0.1	180	Ar	230

**Table 4:** Parameters of the XRD analysis of thin films.

Name	Incident Angle, °	Registration Interval, °	Scan Step, °	Time at Point, Seconds
Specimen 1	3	10–90	0.07	1
Specimen 2	3 and 6	25–100	0.02	3

**Table 5:** Results of EDX analysis of specimen 1.

Atomic Concentrations of the Elements, at.%				
Co	Cr	Fe	Ni	Ti
25.2	24.9	25.5	19.8	4.6

**Table 6:** Results of AES analysis of specimen 1.

Atomic Concentrations of the Elements, at.%					
Co	Cr	Fe	Ni	Ti	O
25.17	26.73	21.8	18.95	3.42	3.93

## Data Availability

Not applicable.
